# Improved Antitumor Activity of a Therapeutic Melanoma Vaccine through the Use of the Dual COX-2/5-LO Inhibitor Licofelone

**DOI:** 10.3389/fimmu.2016.00537

**Published:** 2016-12-05

**Authors:** Silke Neumann, Simon A. Shirley, Roslyn A. Kemp, Sarah M. Hook

**Affiliations:** ^1^School of Pharmacy, University of Otago, Dunedin, New Zealand; ^2^Department of Pathology, Dunedin School of Medicine, University of Otago, Dunedin, New Zealand; ^3^Department of Microbiology and Immunology, University of Otago, Dunedin, New Zealand

**Keywords:** cancer vaccine, melanoma, NSAID, COX-2/5-LO, myeloid-derived suppressor cells, immature myeloid cells, liposomes, α-galactosylceramide

## Abstract

Immune-suppressive cell populations impair antitumor immunity and can contribute to the failure of immune therapeutic approaches. We hypothesized that the non-steroidal anti-inflammatory drug licofelone, a dual cyclooxygenase-2/5-LO inhibitor, would improve therapeutic melanoma vaccination by reducing immune-suppressive cell populations. Therefore, licofelone was administered after tumor implantation, either alone or in combination with a peptide vaccine containing a long tyrosinase-related protein 2-peptide and the adjuvant α-galactosylceramide, all formulated into cationic liposomes. Mice immunized with the long-peptide vaccine and licofelone showed delayed tumor growth compared to mice given the vaccine alone. This protection was associated with a lower frequency of immature myeloid cells (IMCs) in the bone marrow (BM) and spleen of tumor-inoculated mice. When investigating the effect of licofelone on IMCs *in vitro*, we found that the prostaglandin E_2_-induced generation of IMCs was decreased in the presence of licofelone. Furthermore, pre-incubation of BM cells differentiated under IMC-inducing conditions with licofelone reduced the secretion of cytokines interleukin (IL)-10 and -6 upon lipopolysaccharides (LPS) stimulation as compared to untreated cells. Interestingly, licofelone increased IL-6 and IL-10 secretion when administered after the LPS stimulus, demonstrating an environment-dependent effect of licofelone. Our findings support the use of licofelone to reduce tumor-promoting cell populations.

## Introduction

Successful cancer vaccines not only need to stimulate a robust antitumor immune response, but also need to alleviate the effects of immune-suppressive cell populations (1). Inflammatory factors secreted by tumor cells can polarize stromal and immune cells, including macrophages, immature myeloid cells (IMCs) [also known as myeloid-derived suppressor cells (MDSCs)], and T-cells, toward an immune-suppressive phenotype (2). Furthermore, the constant secretion of inflammatory molecules by tumor cells into the systemic circulation promotes IMCs to egress from the bone marrow (BM) and to infiltrate the tumor where they suppress antitumor immunity (3). IMCs have been identified as one of the major pro-tumor immune populations and are positively correlated with melanoma progression, which makes them an attractive target for immunotherapy (4, 5).

Immature myeloid cells are a heterogeneous population of myeloid precursor cells that are present in most cancers and which typically expand during disease progression (3). IMCs are induced by tumor-derived inflammatory factors [e.g., VEGF, granulocyte-macrophage colony-stimulating factor (GM-CSF), prostaglandins, interleukin (IL)-1β, IL-6, IL-10, TGF-β, S100A8, and S100A9] (2, 6, 7) that stimulate tumor growth and myelopoiesis and block the differentiation of IMCs into mature effector cells such as DCs, macrophages, and granulocytes (8, 9). In mouse models, IMCs can be divided into distinct subpopulations based on cell morphology and the expression of the cell surface markers Gr-1 and CD11b, as well as by their suppressive functions (10). The suppressive ability of monocytic IMCs (CD11b^+^ Gr-1^intermediate^) is mediated through an increased catabolism of the essential amino acid arginine, which results in decreased proliferation, anergy, and increased apoptosis of T-cells (2). Granulocytic IMCs (CD11b^+^ Gr-1^high^) produce large amounts of reactive oxygen species (ROS) that impede T-cell receptor signaling (10). Activated IMCs can themselves produce pro-inflammatory stimuli such as IL-6, prostaglandin E_2_ (PGE_2_), and VEGF, which provide a positive feedback loop for their recruitment and activation (8).

One of the key inflammatory factors closely linked to carcinogenesis as well as to the induction, accumulation, and activation of IMCs is PGE_2_ (11). PGE_2_ is a physiologically abundant eicosanoid, derived from arachidonic acid through the cyclooxygenase (COX)-1 and -2 enzymatic pathways (12). PGE_2_ synergizes with leukotriene B_4_ (LTB_4_) to induce local inflammation and to attract immune cells into tissue (13). Leukotrienes (LTs) are also produced from arachidonic acid but require the enzyme 5-lipoxygenase (5-LO) for the production of inflammatory mediators (14). 5-LO expression is detectable in granulocytes, monocytes, and macrophages, which produce the largest quantities of LTs, while expression in DCs, B-cells, and mast cells is lower (15). In contrast to COX-1, which is constitutively expressed in almost all tissues, the expression of COX-2 is tightly regulated. Apart from constitutive expression in the brain and kidneys, COX-2 expression in other tissues is low but can rapidly be induced by inflammatory factors (16). Furthermore, overexpression of COX-2 has been observed in many cancers (13).

Recently, blockade of PGE_2_ using the non-steroidal anti-inflammatory drug (NSAID), celecoxib (a specific COX-2 inhibitor), was shown to improve immunotherapy by inhibiting the generation of IMCs (17). Similarly, the blockage of LTB_4_-mediated effects improved antitumor immunity in a mouse model (18). Since COX-2 and 5-LO convert the same substrate, inhibition of either of these pathways frees up arachidonic acid that can be used by the other enzyme, therefore simultaneous inhibition of both enzymes could offer superior anti-inflammatory effects combined with a better safety profile (19). To date, several NSAIDs with dual COX-2/5-LO activity have undergone clinical testing for use in the treatment of inflammatory diseases, with licofelone (2-[2-(4-chlorophenyl)-6,6-dimethyl-1-phenyl-5,7-dihydropyrrolizin-3-yl]acetic acid) being the most advanced. In addition to demonstrating an excellent tolerability, licofelone has been shown to have a dose-dependent anticancer effect in cell culture and in animal models (20–22). So far studies have focused on the ability of licofelone to directly inhibit PG (21) and LT synthesis (23) and induce apoptosis in tumor cells; however, little is known about its direct effects on immune cell populations in the context of cancer.

We hypothesized that a combination therapy utilizing the NSAID licofelone along with a therapeutic cancer vaccine would decrease inflammatory signaling through COX-2/5-LO inhibition in immune cells and thus facilitate the development of antitumor immunity. To test this hypothesis, licofelone was administered to mice following implantation of tumor cells and was incorporated into a therapeutic liposomal cancer vaccine containing a long peptide and the adjuvant α-galactosylceramide (α-GalCer), previously shown to increase tumor survival in a melanoma model (24). Immunization with long peptides containing both a CD4 and CD8 T-cell epitope induce improved immune responses as compared to minimal T-cell epitopes, which do not require processing prior to presentation on MHC molecules and can induce tolerance (25, 26). The combination of a long peptide [tyrosinase-related protein (TRP) 2], the adjuvant α-GalCer, and licofelone prolonged the survival of tumor-bearing mice as compared to the TRP2–α-GalCer vaccine alone. Further, we show that the prolonged survival correlated with a decrease in the number of IMCs in tumor-bearing mice. Further investigation of the effects of licofelone on IMCs *in vitro* demonstrated changes in IMC phenotype including their ability to produce pro-inflammatory cytokines. These findings support the use of licofelone as an additional component in cancer vaccination regimens, enhancing their immunotherapeutic potential.

## Materials and Methods

### Materials

Lipopolysaccharides (LPS from *Escherichia coli* 055:B5), collagenase from *Clostridium histolyticum* Type IA, phosphatidylcholine (PC), 2′,7′-dichlorofluorescin diacetate (DCFDA), and PGE_2_ were purchased from Sigma Aldrich, USA. Murine IL-6 and -10 CBA flex set were purchased from BD Bioscience, USA. Murine GM-CSF was purchased from Biolegend, New Zealand. The lipids 1,2-dioleoyl-3-trimethylammonium-propane (chloride salt) (DOTAP) and 1,2-dioleoyl-*sn*-glycero-3-phosphoethanolamine (DOPE) were bought from Avanti Polar Lipids, USA. α-GalCer, synthesized from galactose and phytosphingosine, TCI P1765, *via* α-specific glycosylation methodology (27), was kindly provided by Gavin Painter, Ferrier Research Institute, New Zealand. The long TRP2-peptide [TRP2_(180–188),(88–102)_-SVYDFFVWLKFFHRTCKCTGNFA] and the CD4 and CD8 OVA peptides (ISQAVHAAHAEINEAGR and SIINFEKL, respectively) were purchased from Mimotopes, Australia. B16F10luc2 melanoma cells were obtained from ATCC, New Zealand. Licofelone was from AdooQ Bioscience, CA, USA. Carboxyfluorescein diacetate succinimidyl ester (CFSE) was purchased from Molecular Probes, USA. CD43 microbeads were from Miltenyi Biotec, Germany.

### Preparation and Characterization of Cationic Liposomes

Cationic liposomes containing the long TRP2-peptide and αGalCer were prepared by hydrating thin lipid films as described previously (24). For some formulations, 4.43 mg licofelone was added to the lipid precursor prior to hydration. All liposomal formulations were diluted with sterile PBS in order to deliver 200 ng/mouse α-GalCer, 20 nmol/mouse long TRP2-peptide, and 5 mg/kg licofelone per injection. Cationic liposomes containing only 5 mg/kg licofelone were similarly prepared. Particle size (*Z*-average) and polydispersity (PDI) of the preparations were analyzed by photon correlation spectroscopy. Electrophoretic mobility was measured in order to determine the zeta potential of the formulations. Results are the average of three independent samples measured in triplicate at 25°C.

### Mice

The 6- to 10-week-old C57BL/6J and OT-I mice were bred and maintained under specific pathogen-free conditions at the HTRU, University of Otago. The Animal Ethics Committee, University of Otago, approved all experiments (AEC 16/14).

### Tumor Challenge

B16F10luc2 melanoma cells (1 × 10^5^ cells) were injected subcutaneously into the right flank C57BL/6 mice and tumor growth and body weight were monitored every 2 days. Tumor size was assessed by measuring the length and the width of the tumor using digital calipers and mice were culled when tumors reached predetermined humane end points or at 20 days post-tumor injection.

### Immunization Schedule

B16F10luc2 melanoma-bearing mice were randomly divided into five groups of seven mice each and were left untreated or were injected intravenously on day 6 with vaccine (TRP2-peptide and α-GalCer in cationic liposomes) or licofelone-vaccine (TRP2-peptide, α-GalCer, and licofelone in cationic liposomes) plus additional licofelone in cationic liposomes or with only the licofelone liposomes (no vaccine). The licofelone liposomes were given by subcutaneous injection in the neck once every second day, starting on the day of tumor grafting until day 14 of the study.

### Phenotyping of Immune Subsets after Tumor Challenges

Mice were sacrificed 20 days after tumor cell injection and single cell suspensions were prepared from the tumor-draining lymph nodes (td-LNs), spleens, and one hind limb (for the recovery of BM cells). BM was extracted from the hind limbs as described previously (24). The cells were re-suspended at 1 × 10^6^ cells/mL and incubated with the following antibodies after non-specific binding was blocked by incubation with Fc receptor block (F16/32): CD11b PE-Cy7, Gr-1 FITC, CD11c APC, F4/80 brilliant violet 421, and CD3 APC-Cy7 (all antibodies were purchased from Biolegend, New Zealand and were titrated on splenocytes and BM cells). Propidium iodide (PI) was added prior to acquiring data using a BD FACSCanto II and FlowJo software (version 9.5.2, TreeStar, Inc., USA) was used to analyze data.

### Viability and ROS Production of *Ex Vivo*-Derived Melanoma Cells

Macerated tumors from C57BL/6 mice were incubated with collagenase from *C. histolyticum* Type IA (1 mg/mL) and calcium chloride (44.1 μg/mL) at 37°C for 30 min. Red blood cells were lysed and the tumor cells were re-suspended at 5 × 10^5^ cells/mL in cIMDM and seeded into a 24 well plate. These cells were incubated with increasing doses of licofelone (1–20 μM) for 48 h and viability and ROS production were examined by flow cytometry after incubation with PI and 1 μL of freshly prepared DCFDA (10 μM stock solution).

### Generation and Phenotyping of IMCs

Bone marrow cells were isolated from naïve C57BL/6 mice as described previously (28, 29) and seeded at 5 × 10^5^ cells/mL in the presence of GM-CSF (20 ng/mL) and PGE2 (9.1 μg/mL). Increasing concentrations of licofelone (2.5–50 μM) were added to the culture and refreshed every other day (11) together with media and cytokines. Cells were harvested on day 5 of culture and the phenotype analyzed. In some experiments, BM cells harvested from tumor-bearing mice and were re-cultured for 24 h in the presence of either licofelone (5 μM), LPS (50 ng/mL), or a combination of both. Culture supernatants were stored at −20°C for subsequent cytokine analysis using BD Biosciences CBA Mouse Flex Sets. Samples were run on a BD FACSCanto II and the FCAP Array software (v1.0, Soft Flow) was used to calculate cytokine concentrations in the samples and the standards.

### T-Cell Suppression Assay

The suppressive potential of IMCs was evaluated by their ability to inhibit antigen-specific T-cell proliferation. To prepare target T-cells, spleens were dissected from naive OT-I mice and CD43^+^ cells were isolated by positive selection on an AutoMACS Pro Separator (Miltenyi Biotec) to exclude B-cells. CFSE labeled (24) CD43^+^ splenocytes were seeded at 5 × 10^4^ cells/well in a 96 well round-bottom plate and stimulated with the CD8 epitope of ovalbumin (SIINFEKL; 0.01 μg/mL) in the presence or absence of IMCs for 48 h. IMCs were generated *in vitro* as described above and incubated with licofelone (5 μM) for 20 h before LPS (50 ng/mL) was added for 4 h. Cells were washed, re-suspended, and added to the sorted splenocytes at the indicated ratios. After 48 h the proliferation of T-cells was assessed by flow cytometry.

### Statistical Analysis

The statistical significance between values was compared using one-way ANOVA followed by *post hoc* Tukey’s pairwise comparison. All data are expressed as the mean + SD. Survival curves were compared by using the Log-rank test (Mantel–Cox). Statistical analysis was performed using GraphPad Prism version 6.00. All experiments were repeated at least twice.

## Results

### Incorporation of the NSAID Licofelone into Therapeutic Cancer Vaccine Regimens Improves Survival of Tumor-Bearing Mice

We have recently shown that a therapeutic vaccine, consisting of a long TRP2-peptide co-delivered with α-GalCer in cationic liposomes, increased cytotoxic T-cell responses, and improved tumor survival in melanoma-bearing mice (24). To investigate if the antitumor efficacy of the vaccine could be enhanced, the dual COX-2/5-LO inhibitor licofelone was added to the therapy. Licofelone was included as part of the vaccine and was also delivered by subcutaneous injection pre- and postimmunization, as we hypothesized that this would allow enhanced T-cell priming.

We characterized the cationic liposomes containing the long TRP2-peptide, α-GalCer ± licofelone regarding their size, PDI, and zeta potential as the size and charge of liposomes influences their immunogenicity (25). Cationic liposomes containing the long TRP2-peptide and α-GalCer displayed a size of 280 ± 87 nm and a positive surface charge of 43.6 ± 0.8 mV (Table 1). Incorporation of licofelone into cationic liposomes, together with the TRP2-peptide and α-GalCer, did not markedly alter the size (243 ± 43 nm) or surface charge of the cationic liposomes. However, liposomes containing only licofelone alone were significantly larger (802 ± 59 nm, *p* < 0.001).

**Table 1:** **Overview of average particle size, PDI index, and zeta potential of vaccine formulations**.

Cationic liposomes containing	*Z*-average (nm)	PDI	Zeta potential (mv)
Long TRP2-peptide + α-GalCer	280 ± 87	0.34 ± 0.2	43.6 ± 0.8
Long TRP2-peptide + α-GalCer + licofelone	226 ± 43	0.25 ± 0.1	42.5 ± 2.6
Licofelone	802 ± 59	0.33 ± 0.1	39.9 ± 14.5

**Figure 1:**
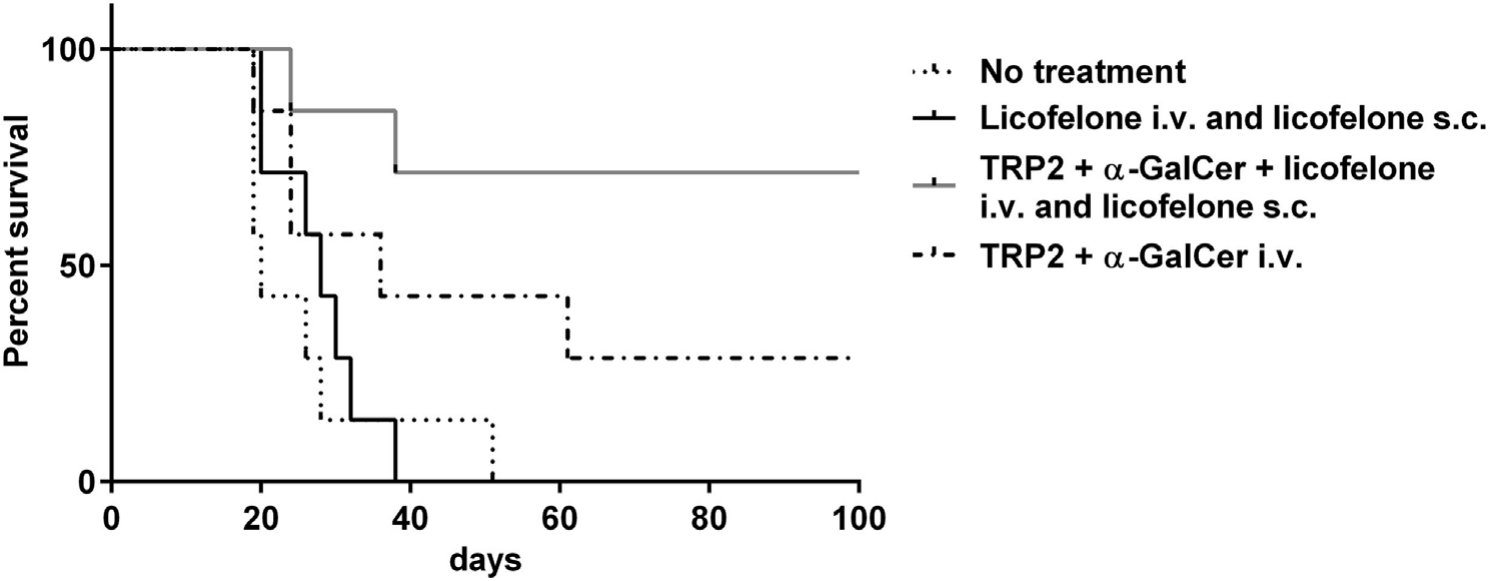
**Addition of licofelone to a long-peptide cancer vaccine therapy prolongs survival of melanoma-bearing mice**. Groups of mice (*n* = 7) were injected s.c. with 5 × 10^4^ B16F10luc2 tumor cells and were subsequently left untreated or injected i.v. on day 6 with TRP2-peptide and α-GalCer in cationic liposomes or TRP2-peptide, α-GalCer, and licofelone in cationic liposomes. Mice that received a vaccine containing licofelone in cationic liposomes were also injected s.c. every second day with the licofelone formulated into cationic liposomes while a control group of mice received only s.c. injections of licofelone liposomes. The graph shows the Kaplan–Meier survival curve. Data are representative of two independent experiments with each *n* = 7 mice/group, ***p < 0.0001.

Tumor-bearing mice were treated i.v. with the liposomal vaccine containing the long TRP2-peptide, α-GalCer ± licofelone, or with liposomal licofelone alone. In addition, groups immunized i.v. with the vaccine containing licofelone, or liposomal licofelone alone, received repeated s.c. injections of liposomal licofelone. Immunization with the long TRP2-peptide vaccine prolonged the survival of melanoma-bearing mice compared to untreated mice, with the best outcome being seen when mice were also given licofelone. Vaccination with the combination of long TRP2-peptide vaccine and licofelone resulted in 5/7 (71.4%) of the vaccinated mice remaining tumor free (*p* < 0.01) (Figure 1). Mice injected with licofelone had the same survival times as untreated mice. No adverse effects were observed with vaccine treatments, as indicated by steady body weight over the vaccine treatment period (data not shown).

### Treatment with Licofelone-Containing Vaccine Decreases Gr-1^+^ CD11b^+^ Cell Population in Tumor-Inoculated Mice

**Figure 2:**
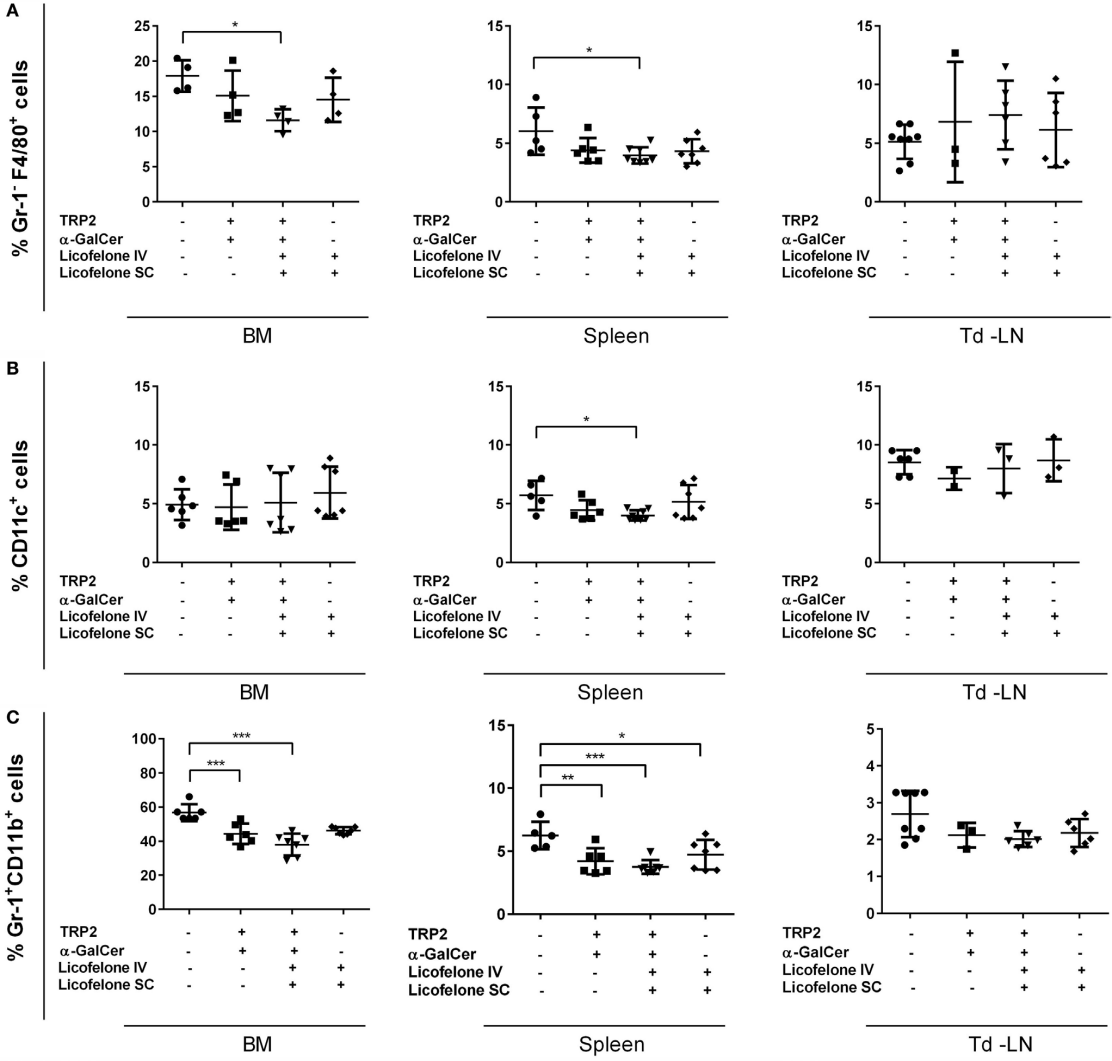
**Addition of licofelone to the vaccine therapy reduces the frequency of myeloid cell populations in the BM and the spleens of tumor-bearing mice**. Mice were manipulated as described in Figure 1 and the BM, spleens, and td-LNs were collected on day 20 and analyzed for the frequency of **(A)** Gr-1^−^ F4/80^+^ macrophages, **(B)** Gr-1^+^ CD11b^+^ IMCs, and **(C)** CD11c^+^ DCs by flow cytometry. Frequencies are shown as a percentage of live cells (PI^low^ cells). Data shown are the mean ± SD of five to seven mice from two experiments. ***p < 0.001, **p < 0.05, *p < 0.01.

We hypothesized that the addition of licofelone prolonged the survival of tumor-bearing mice by reducing the frequency of immune-suppressive populations in lymphatic organs. Therefore, we examined the frequency of myeloid cell populations in the BM, spleen, and td-LNs 20 days after tumor inoculation. Tumor-inoculated mice treated with i.v. licofelone-vaccine plus additional s.c. licofelone showed a decrease in the frequency of Gr-1^−^ F4/80^+^ macrophages and CD11c^+^ cells in spleen and, for Gr-1^−^ F4/80^+^ macrophages, in BM (Figure 2). Examination of myeloid subsets in the td-LNs showed a trend toward a reduced frequency of Gr-1^+^ CD11b^+^ IMCs in mice treated with any of the three formulations; however, this was not significant.

The frequency of Gr-1^+^ CD11b^+^ IMCs in the BM and the spleen was reduced in mice treated with either the i.v. vaccine or the i.v. licofelone-vaccine plus additional s.c. licofelone. In addition, the frequency of Gr-1^+^ CD11b^+^ IMCs in spleen was reduced after licofelone treatment alone (i.v. licofelone plus s.c. licofelone), without vaccination with the long TRP2-peptide and α-GalCer. No significant changes were observed in the frequency of CD4^+^ and CD8^+^ T-cell populations (data not shown).

### Licofelone Does Not Inhibit Melanoma Cell Growth and ROS Production *In Vitro*

**Figure 3:**
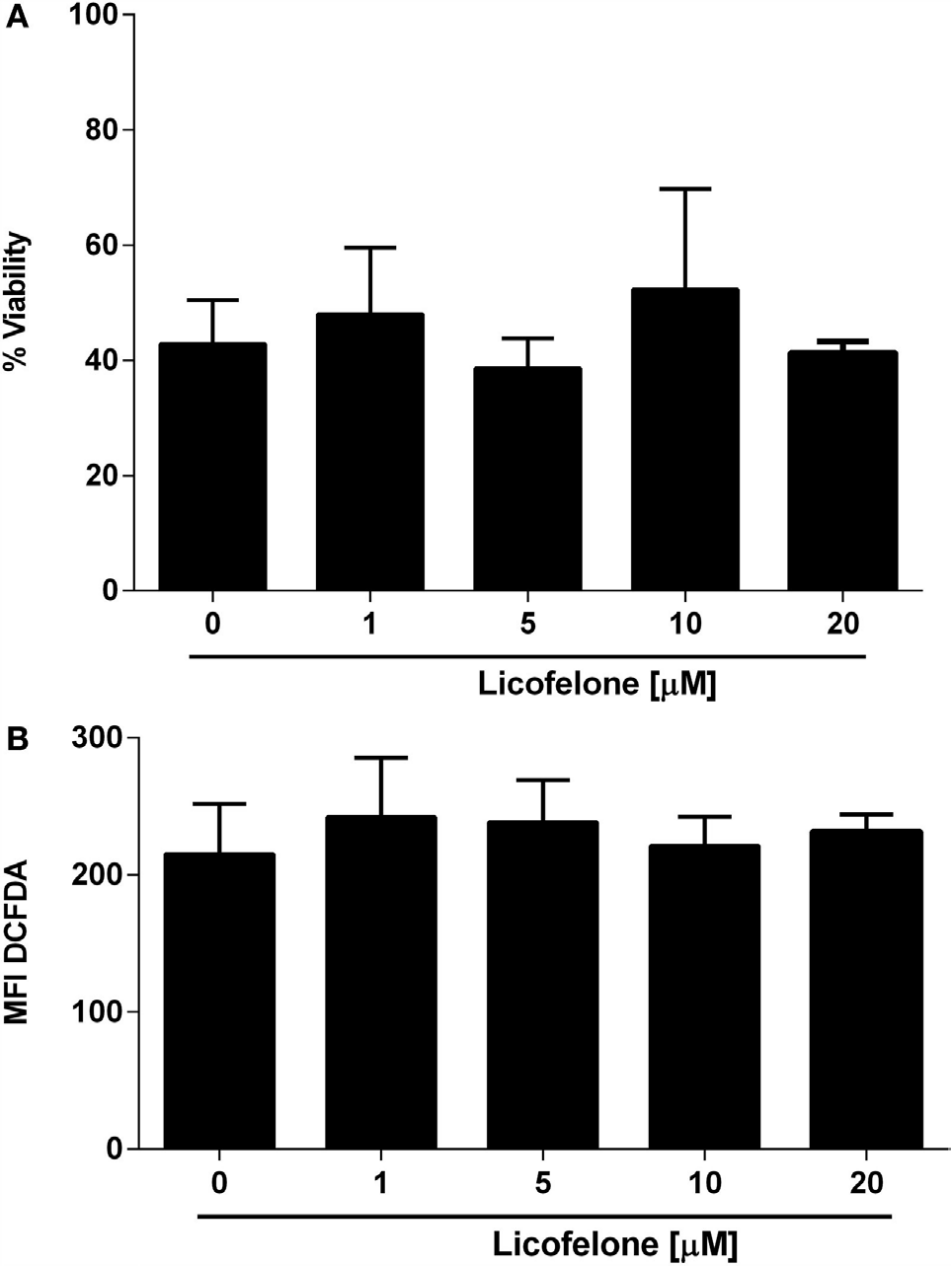
**Viability (A) and ROS production (B) of melanoma cells derived *ex vivo* are not changed by licofelone treatment**. B16F10luc2 melanoma cells were treated with licofelone at 1, 5, 10, or 20 μM for 48 h. Data shown are the mean + SD from three individual experiments.

In order to examine if the antitumor effect of licofelone observed *in vivo* was attributable to a direct inhibition of tumor cell growth, we evaluated the viability of B16F10luc2 melanoma cells, derived *ex vivo*, after a 48-h incubation with licofelone. No significant reduction in viability was observed after incubation of B16F10luc2 melanoma cells harvested from tumor-bearing mice with licofelone at 1, 5, 10, or 20 μM (Figure 3A). Similarly, the production of ROS by melanoma cells, as measured by DCFDA staining, was not changed after treatment with licofelone (Figure 3B).

### Treatment of Gr-1^+^ CD11b^+^ IMCs with Licofelone Reduces the Production of IL-6 and -10 in a Time-Dependent Manner

Increased generation of IMCs in the BM and subsequent accumulation within the tumor has been associated with cancer progression (30). Since licofelone affected the phenotype of BM cells differentiated in the presence of inflammatory mediators associated with tumor progression, we examined if licofelone also influenced the secretion of pro-inflammatory cytokines by IMCs. IL-6 and IL-10 are important inflammatory mediators involved in Gr-1^+^ CD11b^+^ mediated immune suppression. The levels of these two cytokines were analyzed in PGE_2_ and GM-CSF differentiated BM cultures treated either with licofelone (5 μM) and/or LPS (50 ng/mL). LPS was added to activate the cells and induce cytokine production.

**Figure 4:**
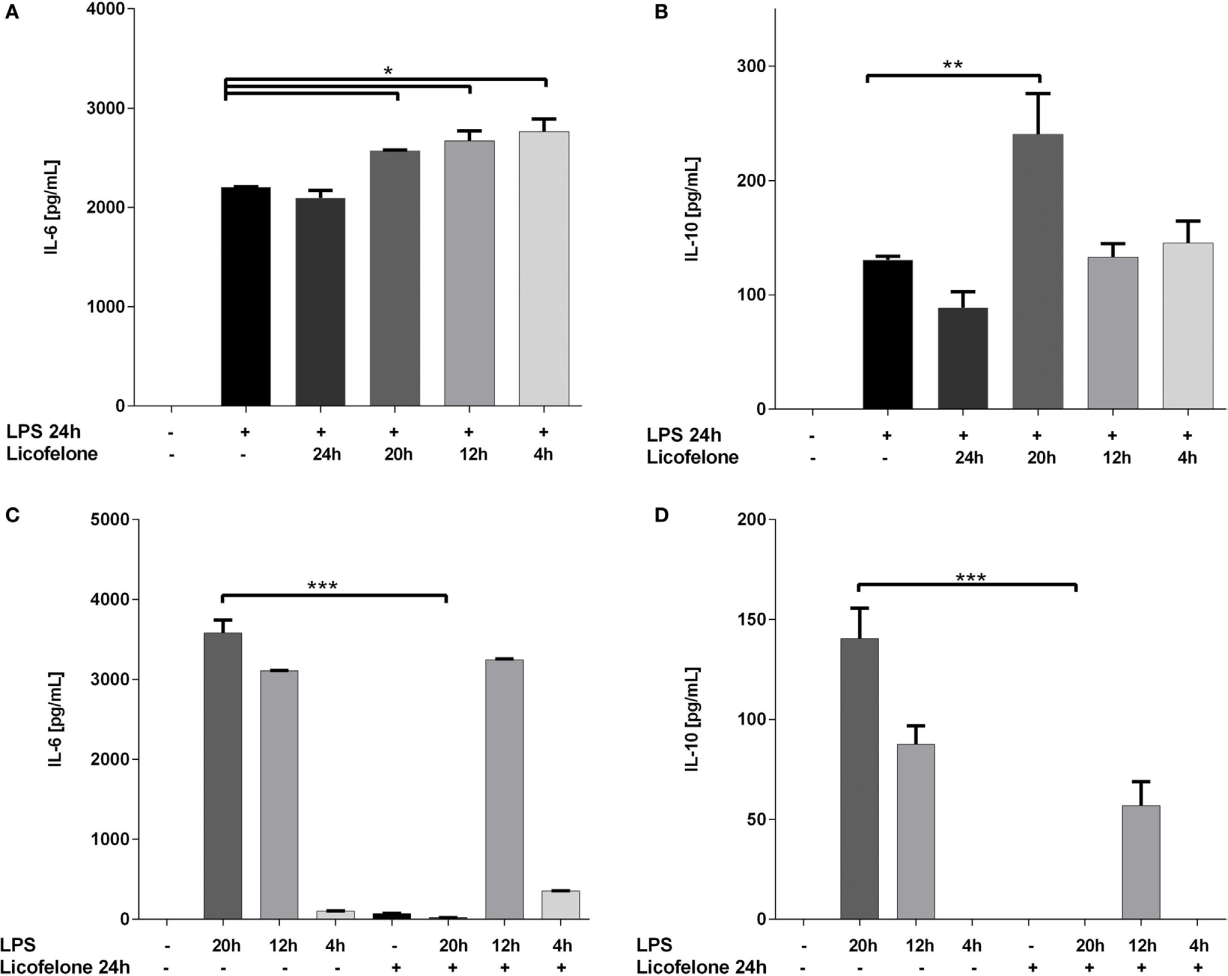
**Licofelone increases IL-6 (A,C) and IL-10 (B,D) production when added to LPS-stimulated cells but decreases the production of the two cytokines when added prior to LPS stimulation**. IMCs generated from the BM of tumor-bearing mice with GM-CSF (20 ng/mL) and PGE_2_ (9.1 μg/mL) for 5 days. Cells were treated either with licofelone (5 μM) and/or LPS (50 ng/mL) alone as described. Data shown are mean + SD of pooled groups from two independent experiments. ***p < 0.0001.

While concentrations of IL-6 increased when licofelone was added to the cultures for as little as 4 h after LPS activation (Figure 4A), the treatment of cells with licofelone and LPS only resulted in a significant increase in IL-10 when cultures were supplemented with licofelone for 20 h after LPS activation (Figure 4B). The reduced levels of IL-10 measured at 24 h may be due to autocrine IL-10 uptake as blocking uptake with anti-IL-10 receptor antibodies has shown to increase the amount of IL-10 detected in culture (31).

The reverse experiment was also carried out whereby the BM cells differentiated under IMC-inducing conditions were pre-incubated with licofelone before the addition of LPS for 4–20 h. Control levels of cytokine in LPS-stimulated cells were measured to assess the effect of licofelone pre-incubation. Treatment of cells with licofelone for 4 h prior to the addition of LPS (for the remaining 20 h) completely ablated the production of IL-6 and IL-10 (Figures 4C,D). Cells stimulated with LPS at later time points did not result in a decrease in amounts of both IL-6 and IL-10 produced.

### Licofelone Suppresses the Generation of Gr-1^+^ CD11b^+^ IMCs and Gr-1^−^ F4/80^+^ Macrophages *In Vitro*

**Figure 5:**
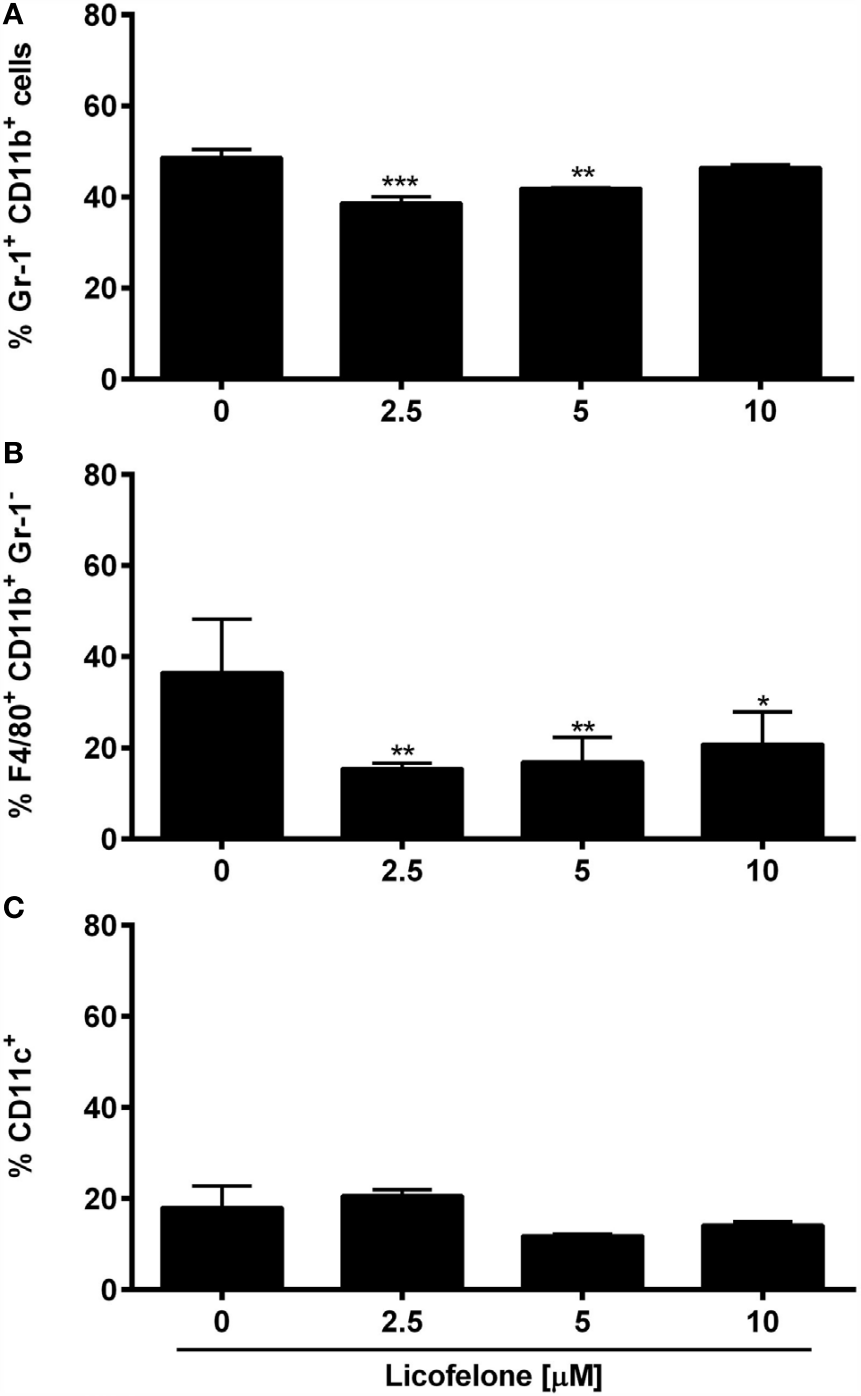
**Licofelone affects the generation of IMCs and macrophages *in vitro***. BM cells were harvested from the femur of naive C57BL/6 mice and were incubated with media and GM-CSF (20 ng/mL), PGE_2_ (9.1 μg/mL), and licofelone (2.5, 5, and 10 μM) for 5 days. The frequency of **(A)** Gr-1^+^ CD11b^+^ IMCs, **(B)** Gr-1^−^ F4/80^+^ macrophages, and **(C)** CD11c^+^ DCs was determined by flow cytometry. Frequencies are shown as a percentage of live cells (PI^low^ cells). The results are the mean + SD of three independent experiments (*n* = 2/experiment). ***p < 0.001, **p < 0.01, *p < 0.05.

The COX-2 inhibitor celecoxib (32) and several E-prostanoid receptor antagonists (11) have been shown to modulate the generation and the suppressive potential of IMCs. To investigate if the dual COX-2/5-LO inhibitor licofelone shapes myeloid cell populations, BM cells were cultured in the presence of inflammatory factors (PGE_2_ and GM-CSF) commonly secreted by tumor cells (11) and increasing doses of licofelone. The generation of Gr-1^+^ CD11b^+^ IMCs was significantly suppressed in the presence of 2.5 and 5 μM licofelone while the generation of Gr-1^−^ F4/80^+^ macrophages was suppressed with all three concentrations of licofelone used (Figure 5). The addition of licofelone had no impact on the generation of CD11c^+^ cells.

### Licofelone Attenuates the Suppressive Function of Gr-1^+^ CD11b^+^ IMCs

**Figure 6:**
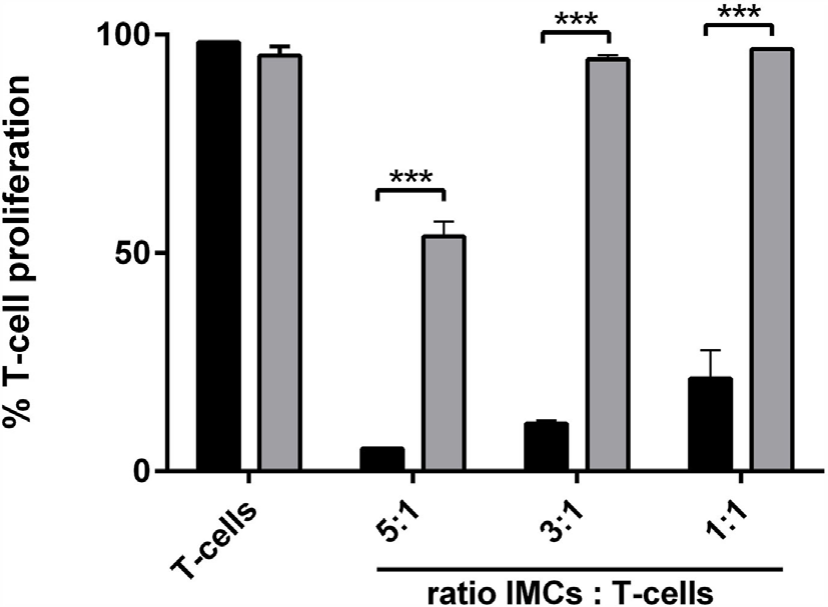
**Incubation with licofelone reduces suppressive activity of Gr-1^+^ CD11b^+^ IMCs**. BM cells differentiated in the presence of GM-CSF (20 ng/mL) and PGE_2_ (9.1 μg/mL) were incubated with licofelone (5 μM) for 20 h (gray bar) or media (black bar) and then pulsed with LPS and OVA_257–264_ for 4 h before they were cocultured with CFSE-stained splenocytes from OT-I mice in varying ratios. After 72 h, the proliferation of OVA stimulated T-cells was assessed by flow cytometry. Data are representative of three independent experiments with two replicates + SD each, ***p < 0.0001.

Next, we examined if licofelone was able to ameliorate or reverse the suppressive function of CD11b^+^ Gr-1^+^ IMCs *in vitro*. CD11b^+^ Gr-1^+^ IMCs were generated *in vitro* from BM precursor cells, pulsed with the SIINFEKL peptide, and activated with LPS prior to coculture with OT-I mouse splenocytes. The proliferation of T-cells was suppressed at all investigated ratios with licofelone being able to reverse the suppressive function of CD11b^+^ Gr-1^+^ IMCs partially (Figure 6).

## Discussion

One of the biggest obstacles preventing the success of cancer vaccines is the ability of tumor cells to evade killing by the immune system. This is facilitated by the development of immune-suppressive cell populations in the BM, which subsequently migrate to the tumor. Here we investigated the potential of the NSAID licofelone, a dual COX-2/5-LO inhibitor, to reduce the frequency of immune-suppressive cells in lymphatic organs and to stimulate antitumor immunity when used in combination with a cancer vaccine. Licofelone was administered in two ways, once i.v. on day 6 after tumor grafting with the other vaccine components (long TRP2-peptide and α-GalCer) in cationic liposomes, and also every second day s.c. over a period of 14 days beginning on the day of the tumor grafting, again formulated into cationic liposomes.

Previous reports have described a chemo-preventive effect of licofelone in colon cancer (20) and lung adenoma (22, 33) models when administered directly to the cancer site. While *in vitro* low concentrations (2.5–40 μM) of licofelone decreased proliferation of several prostate tumor cell lines by inducing apoptotic cell death (21), at higher concentrations (150 μM) licofelone induced mitochondrial dysfunction (34). However, we found no effect of systemic licofelone monotherapy on the development of solid tumors in the melanoma model and no direct effect of licofelone on the viability of B16F10luc2 tumor cells harvested from tumor-bearing mice *in vitro*. Similarly, licofelone did not influence the ROS production of B16 melanoma cells isolated from tumor-bearing mice. These results align with studies that demonstrate COX-2 independent inhibition of ROS production by chemo-preventive drugs, such as nitric oxide-donating aspirin, and suggest that these are off-target effects of the COX inhibitor (35). Together this indicates that in this model the NSAID needs to be given in combination with a vaccine in order to stimulate effective antitumor responses and improve survival. Experiments replacing licofelone with the COX-2 inhibitor celecoxib (data not shown) did not improve survival, suggesting that dual inhibition of both COX-2 and 5-LO is beneficial.

We chose to package the vaccine components and licofelone into cationic liposomes as they are an excellent delivery system for poorly water-soluble drugs, such as licofelone, and are inherently immune-stimulatory (36). The size and charge of the liposomes strongly affects their systemic distribution as well as the uptake and activation of APCs. The positive charge facilitates interactions with the negatively charged cell membranes and is thereby thought to enhance antigen delivery to DCs (37). Furthermore, cationic liposomes activate pro-inflammatory and pro-apoptotic pathways that lead to the generation of ROS (38), enhanced expression of costimulatory molecules such as CD80 and CD86, and secretion of chemokines and cytokines (39), which together should support the generation of an antitumor response. We have previously shown that immunization with a liposomal vaccine, with a similar size and charge as reported here, was superior in terms of eliciting CD8^+^ T-cell responses as compared to immunization with the vaccine components alone (24).

When we analyzed the composition of cells after immunization with the various vaccines, we detected significant differences in the frequency of myeloid cell populations in the spleens and BM of tumor-inoculated mice treated with a licofelone-containing vaccine with the levels of Gr-1^+^ CD11b^+^ IMCs and Gr-1^−^ F4/80^+^ macrophages being decreased. A similar trend was observed in the td-LNs; however, these differences were not statistically significant which is likely due to variation in the size of tumors on the day they were harvested. The frequency of Gr-1^+^ CD11b^+^ IMCs was also decreased in mice treated with the vaccine alone (without licofelone), perhaps indicating that one of the other vaccine components was responsible for this change. For example, the glycolipid α-GalCer, used as an adjuvant in this study, activates iNKT cells, which are known to reduce the suppressive potential of IMCs (40). Moreover, α-GalCer has been shown to promote the maturation of DCs and to contribute to the induction of T-cell responses (41). Even though vaccines containing the iNKT adjuvant α-GalCer improved the survival of tumor-bearing mice, the antitumor effect was further improved by the addition of licofelone to the treatment regimen, significantly extending the median survival time.

The effect of licofelone on myeloid cells from immunized tumor-inoculated mice *ex vivo* and directly *in vitro* was examined. An important factor to consider when interpreting the results from the *in vitro* experiments is the phenotype of cells generated in the chosen culture conditions. Approximately 60–70% of BM-derived cells generated in the presence of GM-CSF express high levels of CD11c and MHCII and are able to stimulate T-cell proliferation in response to antigens (42). The addition of PGE_2_ and IL-4 has been shown to reduce the CD11c^+^ MHCII^+^ population and instead increases the frequency of Gr-1^+^ CD11b^+^ IMCs, which are able to suppress T-cell proliferation (11). In accordance with these studies, we found approximately half of our cells, cultured with GM-CSF and PGE_2_ for 5 days, expressed Gr-1 and CD11b and were able to suppress T-cell proliferation. The addition of licofelone at 2.5–5 μM was able to directly suppress the generation of Gr-1^+^ CD11b^+^ IMCs and Gr-1^−^ F4/80^+^ macrophages. This supports the results of the *ex vivo* analysis where the frequency of Gr-1^+^ CD11b^+^ IMCs and Gr-1^−^ F4/80^+^ macrophages was similarly reduced after licofelone treatment. While there is little other published information regarding dual COX-2/5-LO inhibitors; the COX-2 inhibitor celecoxib, when used in a mesothelioma mouse model, affected both the number and the function of IMCs by inhibiting PGE_2_ synthesis and decreasing ROS and NO production (43). The *in vitro* observed effect of licofelone on macrophages is intriguing as cytokines produced by macrophages upon exposure to PGE_2_, such as IL-6, are important modulators of IMC function (11). Since we observed an effect of licofelone on both, macrophages and IMCs, it remains to be determined if only one of these populations or both are responsible for the observed antitumor immunity. However, the expression of the same cell surface markers by both MDSCs and macrophages makes it difficult to distinguish between the cell populations and consequently the effects of licofelone. For example, an immune-suppressive subset of MDSCs with a low expression of Gr-1 has been described and found to also express F4/80 (44). Since both cell populations are able to suppress T-cell responses, the inhibition of their development by licofelone is advantageous.

Reduced COX-2 and 5-LO activity has also been associated with a decrease in pro-inflammatory cytokines detectable in the serum of tumor-bearing mice. This included IL-6, which is one of the major cytokines involved in the expansion of Gr-1^+^ CD11b^+^ IMCs (20, 45). Therefore the production of IL-6 and IL-10 [which activates the immune-suppressive and tumor-promoting effects of Gr-1^+^ CD11b^+^ IMCs *via* STAT3 and STAT6 signaling (46–48)] by licofelone-treated cells differentiated under IMC-inducing conditions was examined. We found that licofelone treatment of IMC-differentiated LPS-activated cells was unable to reduce production of both IL-6 and IL-10 and instead increased cytokine production in some conditions. However, when licofelone was given before LPS activation, both IL-6 and IL-10 production was almost completely suppressed. Interestingly, the effect of licofelone was transient with suppression only being apparent when licofelone was given around the time of LPS activation. Blidner et al. have shown a differential response of IMCs to the COX-1/2 inhibitor, indomethacin, depending on the IMC microenvironment (49). Under inflammatory conditions, associated with the tumor progression, indomethacin counteracted the suppressive actions of the IMCs and increased their expression of Gr-1. Interestingly, indomethacin treatment in a non-inflammatory environment led to an increase in NO and ROS production in IMCs, amplifying their immune suppression (49, 50). The changes in IL-6 and -10 production after licofelone treatment of LPS-stimulated and unstimulated of Gr-1^+^ CD11b^+^ IMCs complement these observations.

Immune suppression, the key feature of IMCs, is mediated through various metabolic and signaling pathways, as well as through direct cell-to-cell contact (8). Therefore, it was of interest to see that licofelone pre-treatment of BM cells differentiated under IMC-conditions could partially reverse CD8 T-cell suppression. This is in agreement with findings from studies that have found other anti-inflammatory agents, for example celecoxib, to reverse IMC induced T-cell suppression *in vitro* (32, 43, 51). It remains to be determined if the antitumor activity of licofelone solely depends on the inhibition of Gr-1^+^ CD11b^+^ IMCs numbers and function through effects on prostaglandin and LT synthesis; or if other factors, such as inhibition of VEGF production (21), are involved. However utilizing a safe, small molecule drug such as licofelone to reduce tumor-induced immune suppression and potentiate immune responses stimulated by therapeutic vaccines provides an attractive alternative to the use of checkpoint blockade inhibitors; which while proving to be very effective in some situations (52, 53) are expensive, cannot be delivered orally and have the potential for serious immune-related adverse events (53, 54).

## Author Contributions

All the authors reviewed and approved the final version of the manuscript and agreed to be accountable for the content of the work. SN and SS designed the experiments; acquired, analyzed, and interpreted the data; prepared and critically revised the manuscript; and are accountable for all aspects of the work. RK and SH designed the experiments, interpreted the data, critically revised the manuscript, and are accountable for all aspects of the work.

## Conflict of Interest Statement

The authors declare that the research was conducted in the absence of any commercial or financial relationships that could be construed as a potential conflict of interest.

## Supplementary Material

Figure S1 **Gating strategy for identification of IMCs harvested from tumor-bearing mice**. All data collected were analyzed, and doublets were excluded by using forward scatter height and area (FSC-H/FSC-A). Live cells were detected by gating on PI^low^ cells based on the unstained control. The frequency of macrophages was determined by gating on Gr-1^−^ and F4/80^+^ cells. Gating from PI^low^ cells, the gate for CD11c^−^ cells was determined in comparison to an unstained sample. From there, IMCs were identified by the expression Gr-1 and CD11b. The gate for IMCs was chosen in comparison to the relevant FMO.
