# Supplementary figures and images for: Improved Antitumor Activity of a Therapeutic Melanoma Vaccine through the Use of the Dual COX-2/5-LO Inhibitor Licofelone
Source: Front Immunol. 2016 Dec 5;7:537. doi: 10.3389/fimmu.2016.00537 (PMC5137024; doi:10.3389/fimmu.2016.00537)

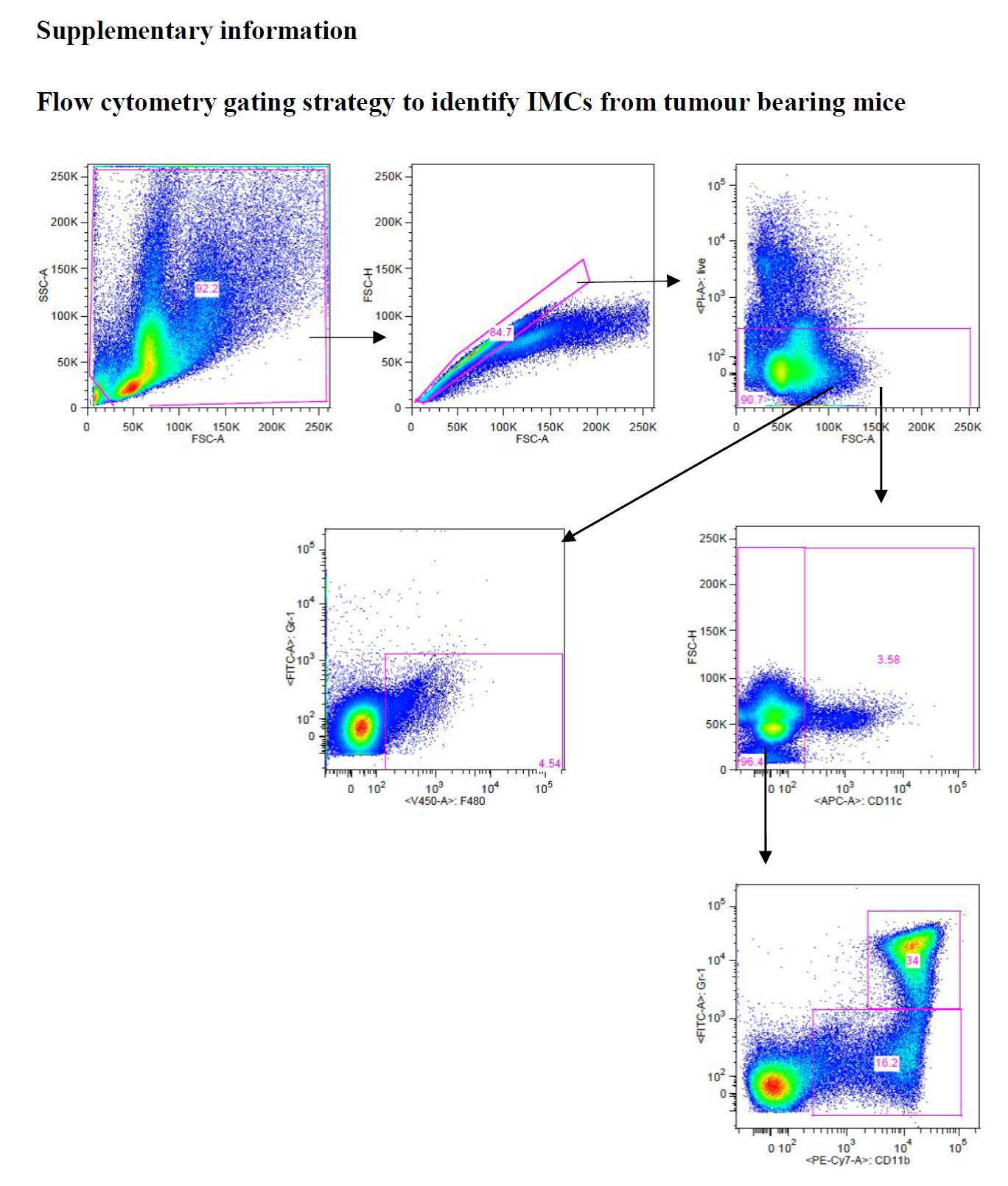

Supplement: Figure S1 — Gating strategy for identification of IMCs harvested from tumor-bearing mice. All data collected were analyzed, and doublets were excluded by using forward scatter height and area (FSC-H/FSC-A). Live cells were detected by gating on PIlow cells based on the unstained control. The frequency of macrophages was determined by gating on Gr-1− and F4/80+ cells. Gating from PIlow cells, the gate for CD11c− cells was determined in comparison to an unstained sample. From there, IMCs were identified by the expression Gr-1 and CD11b. The gate for IMCs was chosen in comparison to the relevant FMO. [file Image_1.JPEG]
